# The role of neutrophils in the dysfunction of central nervous system barriers

**DOI:** 10.3389/fnagi.2022.965169

**Published:** 2022-08-11

**Authors:** Bruno Santos-Lima, Enrica Caterina Pietronigro, Eleonora Terrabuio, Elena Zenaro, Gabriela Constantin

**Affiliations:** Section of General Pathology, Department of Medicine, University of Verona, Verona, Italy

**Keywords:** neutrophils, blood-brain barrier, blood-cerebrospinal fluid barrier (BCSFB), neurodegenerative diseases, neuroinflammation

## Abstract

Leukocyte migration into the central nervous system (CNS) represents a central process in the development of neurological diseases with a detrimental inflammatory component. Infiltrating neutrophils have been detected inside the brain of patients with several neuroinflammatory disorders, including stroke, multiple sclerosis and Alzheimer’s disease. During inflammatory responses, these highly reactive innate immune cells can rapidly extravasate and release a plethora of pro-inflammatory and cytotoxic factors, potentially inducing significant collateral tissue damage. Indeed, several studies have shown that neutrophils promote blood-brain barrier damage and increased vascular permeability during neuroinflammatory diseases. Recent studies have shown that neutrophils migrate into the meninges and choroid plexus, suggesting these cells can also damage the blood-cerebrospinal fluid barrier (BCSFB). In this review, we discuss the emerging role of neutrophils in the dysfunction of brain barriers across different neuroinflammatory conditions and describe the molecular basis and cellular interplays involved in neutrophil-mediated injury of the CNS borders.

## Introduction

The central nervous system (CNS) is physically separated from the peripheral milieu by specialized barriers ensuring a constant state of homeostasis and efficient neuronal function. It was previously considered an immune-privileged site, and this view was attributed to the low number of CNS immune cells and the presence of physical barriers limiting the communication between the systemic circulation and neural tissue (Bechmann et al., 2007; Galea et al., 2007). Over the past decades, this view has been challenged by several studies showing the presence of immune cells, including T and B lymphocytes, in the healthy brain (Anthony et al., 2003; Smolders et al., 2018). Additionally, recent reports describing CNS lymphatics, meningeal tissue and skull bone marrow as active immune hubs contributed to a broader vision of the immune cell dynamics within the healthy CNS (Louveau et al., 2015; Ribeiro et al., 2019; Alves de Lima et al., 2020; Brioschi et al., 2021; Cugurra et al., 2021; Herz et al., 2021; Rustenhoven et al., 2021). Particularly, under physiological conditions, meningeal immunity plays a key role in modulating cognition and behavior in a cytokine-dependent manner, suggesting that brain borders play an active role in brain functions (Filiano et al., 2016; Ribeiro et al., 2019; Alves de Lima et al., 2020; Herz et al., 2021). During CNS inflammatory pathologies, peripheral immune cells migrate into the brain and meninges and play a pathogenic role in several neuropathological disorders (Prinz and Priller, 2017; Rossi et al., 2021). Neutrophils constitute the most abundant immune cell population in the human peripheral blood (Juul et al., 1984), and their role in disease development has been shown in several CNS disorders such as stroke, multiple sclerosis (MS) and Alzheimer’s disease (AD) (Rossi et al., 2020, 2021). Their vast granule storage of enzymes potentially inducing collateral tissue damage, the capacity to rapidly extravasate and increase vascular permeability and the ability to favor tissue remodeling, suggest neutrophils may have a key role in the development CNS inflammatory disorders (Rossi et al., 2020; Fischer et al., 2022). This review summarizes recent data on the contribution of neutrophils to brain injury focusing on pathological mechanisms occurring at CNS borders. We discuss what is known about the involvement of neutrophils in the induction of endothelial injury, increased vascular permeability and degradation of extracellular matrix promoting neuroinflammation and neurodegeneration.

## Overview of the central nervous system barriers

**FIGURE 1:**
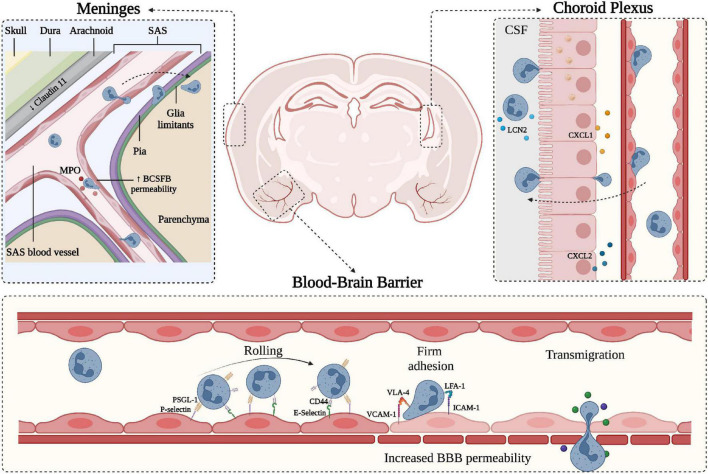
Schematic representation of neutrophil migration through the CNS barriers. CNS barriers limit the accessibility of circulating cells and molecules and can be divided into two main components: the Blood-Brain-Barrier (BBB) situated within the brain parenchyma and the blood-CSF-barrier (BCSFB) present in the choroid plexus (ChP) and in the meninges. Starting from the outer part of the CNS (depicted on the top left), the meninges represent a series of connective layers surrounding the brain, including dura mater and leptomeninges (arachnoid and pia mater). During CNS inflammatory diseases, neutrophils adhere in pial vessels and migrate into the SAS; these cells may then cross pia matter and migrate into the underlying brain parenchyma. Within the leptomeningeal vessels, neutrophils release MPO, potentially damaging endothelial cells and the extracellular matrix. On the top right, a representation of the choroid plexus shows the production of neutrophil chemoattractants CXCL1 and CXCL2 by the epithelial cells leading to neutrophil infiltration across the BCSFB. In both ChP stroma and within the CSF, neutrophils have been shown to produce LCN2. On the lower panel, a simplified model of the BBB is depicted, along with the classical neutrophil migration cascade. Inflamed BBB endothelial cells express adhesion molecules such as P- and E-selectins mediating capture and rolling through interaction with neutrophil PSGL-1 and CD44. Endothelial ICAM-1 and VCAM-1 mediate the arrest phase by binding neutrophil integrins LFA-1 and VLA-4, respectively. Firm adhesion and transmigration may contribute to increased BBB permeability (created with BioRender.com).

**FIGURE 2:**
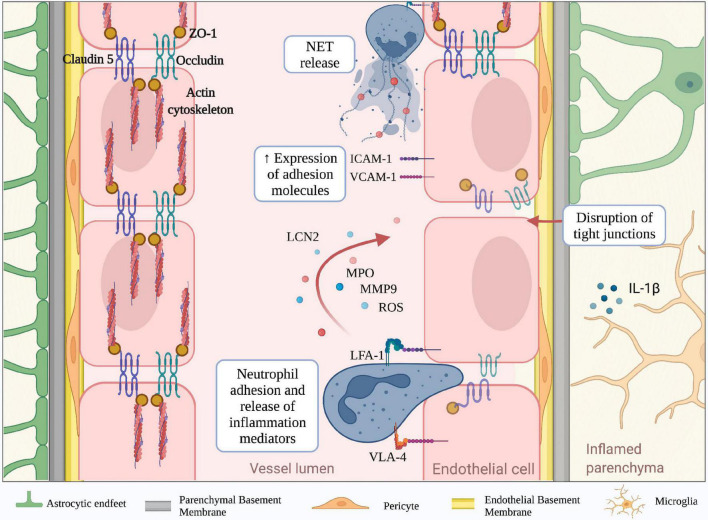
Molecular mechanisms mediating neutrophil-dependent BBB dysfunction. The BBB is a complex structure mainly composed of endothelial cells tightly bound together by junctional molecules, astrocytic endfeet and pericytes. During neuroinflammation, microglia and astrocytes produce IL-1b, which is a potent inducer of endothelial ICAM-1 and VCAM-1 and promotor of neutrophil infiltration. Engagement of neutrophil integrins as well as other stimuli, lead to the production of neutrophil extracellular traps (NETs) and release of inflammatory mediators including reactive oxygen species (ROS), lipocalin 2 (LCN2), myeloperoxidase (MPO) and matrix metalloproteinase 9 (MMP-9). These neutrophil-derived factors contribute to the reduction of tight junction proteins (claudin-5, occludin and ZO-1) and BBB breakdown. (Created with BioRender.com).

The CNS is physically separated from the peripheral milieu by two main barriers: the blood-brain barrier (BBB) and blood-cerebrospinal fluid barrier (BCSFB) (Figure 1). Although both barriers are characterized by high expression of junctional proteins and selective permeability capacities, they also have unique anatomical and molecular characteristics. The BBB is an endothelial structure of brain microvessels, which limits the passage of plasma molecules and blood cells into the brain and maintains the chemical composition of the neuronal “milieu” (Zlokovic, 2008). On the other hand, the BCSFB separates the blood from the CSF and was classically associated with the epithelium of the choroid plexus (ChP) and arachnoid matter (Brøchner et al., 2015; Engelhardt et al., 2017) (Figure 1). Overall, the CNS barriers not only protect the parenchyma, restricting the diffusion of pathogens and harmful blood molecules, but also play a critical role in the uptake of nutrients and excretion of byproducts of brain metabolism (Ballabh et al., 2004). The BBB consists of a monolayer of tightly packed and specialized non-fenestrated endothelial cells (ECs) brought together by junctional complexes, covering brain capillaries and postcapillary venules (Figure 1). It is fenced by the glia limitans formed by perivascular astrocyte endfeet and parenchymal basement membrane, which further restricts BBB permeability. This specialized and dynamic structure, together with the surveying microglia, astrocytes, pericytes, neuronal branches, and the acellular basement membrane, form the neurovascular unit (NVU) (Villabona-Rueda et al., 2019). Furthermore, the BBB represents a high-resistance electrical barrier, expressing transporters and efflux pumps for the regulation of ion fluctuations and solutes, and limiting the paracellular and transcellular movement of molecules. The inter-endothelial junctional structures are responsible for the “gate” function of ECs and consist of tight junction proteins (TJ), adherens junctions (AJs) and gap junctions (Chow and Gu, 2015; Tietz and Engelhardt, 2015; Sweeney et al., 2019; Saint-Pol et al., 2020). Brain endothelial TJs are the most apical cell-cell junctional complexes and constitute the core elements of the BBB with a crucial role in regulating paracellular permeability and maintaining cell polarity. They contain transmembrane proteins, including occludin claudins and junctional adhesion molecules, anchored to intracellular scaffolding proteins of the membrane-associated guanylate kinase family, also known as zonula occludens (ZO-1, -2, -3) (Zlokovic, 2008). The integral membrane proteins provide support to the TJs structure, with occludin contributing to the regulation and stabilization of the junctional structures (Cummins, 2012), whereas claudins are critical sealing determinants of paracellular “tightness” between adjacent ECs (Alahmari, 2021). Particularly, claudin-5, the most abundant transmembrane TJ protein at the BBB, plays a prominent role in maintaining the structural integrity of the vasculature and preventing the diffusion of small molecules through the intercellular space (Figure 2; Greene et al., 2019). Reduced expression of occludin, claudin-5, and ZO-1 is considered a sensitive indicator of BBB alterations associated with increased permeability during CNS diseases (Zlokovic, 2008; Cummins, 2012; Greene et al., 2019). Among the junctional complexes between the cerebral ECs, vascular endothelial (VE)-cadherin represents the major AJ transmembrane protein. VE-cadherin is linked to the cytoskeleton via catenins such as p120, β-catenin, plakoglobin, and γ-catenin, ensuring AJ assembly and BBB stability (Zlokovic, 2008; Li et al., 2018). Besides the organized junctional complexes, additional independent molecules are present at ECs junctions, including platelet-endothelial cell adhesion molecule-1 (PECAM-1 or CD31) and CD99. These proteins have homophilic and heterophilic binding capacity and have a role in the context of leukocyte transmigration during inflammatory responses (Lou et al., 2007; Zlokovic, 2008; Sullivan and Muller, 2014). Alterations in junctional molecules facilitate leukocyte transmigration during brain inflammation. Indeed, the binding of α4β1 integrin expressed on lymphocytes to the endothelial vascular adhesion molecule (VCAM-1) induces the phosphorylation of VE-cadherin and leads to the opening of AJ contacts, facilitating leukocyte transmigration (Vockel and Vestweber, 2013).

The BBB together with the BCSFB are considered part of the neurovascular system, providing active immune surveillance and supporting coordinated immune responses (Engelhardt et al., 2017; Mastorakos and McGavern, 2019). Even though the BCSFB is more accessible and permeable than the BBB, the latter has received more attention for its involvement in CNS pathologies (Felgenhauer, 1995; Tumani et al., 2017). Nevertheless, growing evidence suggests the BCSFB also plays a significant role in the communication between the periphery and the CNS during inflammatory neurological disorders. The BCSFB was mainly associated with two areas of the CNS: the ChP and the arachnoid membrane covering the subarachnoid space (SAS). The ChP is located in the lateral and fourth ventricles and is an epithelial tissue surrounding fenestrated vessels. It represents a major site of CSF production in the brain and strictly regulates the exchange of substances between the blood and the CSF (Lun et al., 2015). ChP capillaries do not have TJs, allowing the free movement of molecules through fenestrations and intercellular gaps. However, the choroidal BCSFB is characterized by the presence of junctional molecules in the epithelial lining of ChP, allowing selective permeability of blood components. Despite functional similarities between the BBB and the BSCFB, the choroidal BCSFB shows mainly the expression of claudins 1–3, 9, 12 and 22 and almost no expression of claudin-5, suggesting morphological differences that may lead to a more permeable barrier to small molecules, macromolecules and circulating immune cells (Kratzer et al., 2012; Tietz and Engelhardt, 2015). The pial microvessel blood-CSF barrier across pial microvessels is also considered part of the BCSFB (Brøchner et al., 2015). Endothelial cells isolated from the pial vessels show similarities with the BBB for some general ultrastructural features and transendothelial electrical resistance (Allt and Lawrenson, 1997). However, pial microvessels show some differences in the junctional systems compared to the BBB and lack astrocyte and NVU proximity, suggesting these vessels are more permeable and easier to cross by leukocytes during inflammatory responses (Allt and Lawrenson, 1997; Cassella et al., 1997).

The meningeal tissue is now considered an active and complex immune hub (Alves De Lima et al., 2020). From a structural point of view, the meninges are divided into three main layers: dura, arachnoid and pia. Dura mater is the outermost layer and is securely attached to the periosteum of the skull (Protasoni et al., 2011). It is composed of a dense and thick fibrous tissue supplied with lymphatic vessels, meningeal arteries and veins (Protasoni et al., 2011). The arachnoid mater is an avascular, thin and translucent membrane forming a barrier between the dura mater and the CSF flowing in the SAS (Yasuda et al., 2013). This space includes trabeculae and collagen bundles generated by fibroblast-like cells, which connect the arachnoid to the pia mater (Alcolado et al., 1988; Saboori and Sadegh, 2015). The deepest layer of the meninges is the pia mater, a thin and transparent membrane composed of connective tissue permeable to solutes and containing a capillary network that nourishes the brain (Adeeb et al., 2013). The pia mater is anchored on the brain surface by astrocyte processes and together with the arachnoid matter forms the leptomeninges (Derk et al., 2021). In addition to their protective role, the meninges also constitute a route of drainage for brain interstitial fluid to lymphatic vessels and deep cervical lymph nodes (Natale et al., 2021), providing communication between the CNS and the immune system. The bona fide barriers from the meningeal tissue can be subdivided into three main interfaces: (1) the BCSFB associated with the arachnoid layer separating the dura and its fenestrated vessels from the CSF; (2) the BCSFB at the level of SAS pial microvessels; and (3) the pial surface layer, which together with glia limitans is considered a brain-CSF barrier (Brøchner et al., 2015). The junctional components of the meningeal barriers are less studied, but recent work identified claudin-11 as a marker for the arachnoid BCSFB (Brøchner et al., 2015). However, further studies are needed to better understand the structural and molecular features of meningeal barriers and how they regulate brain function during health and disease.

## Blood-brain barrier dysfunction during neuroinflammation

Neuroinflammation is a well-defined pathological feature of several neurodegenerative disorders and associates with BBB structural changes and increased vascular permeability (Zlokovic, 2008; Rossi et al., 2011; Zenaro et al., 2017; Liebner et al., 2018). For example, studies performed in stroke models and experimental autoimmune encephalomyelitis (EAE), the most common model of MS, have shown increased BBB permeability due to a reduction of claudin-5, occludin, and ZO-1 levels in the mouse brain (Jiao et al., 2011; Wang et al., 2016). Similar results were obtained in cerebral amyloid-beta angiopathy in AD post-mortem brains, suggesting the reduction of TJ molecules represents a general feature of BBB dysfunction across several brain diseases (Carrano et al., 2012). The redistribution of inter-endothelial junctional proteins during BBB dysfunction depends on their phosphorylation state, which can be affected by growth factors and inflammatory cytokines (Van Itallie and Anderson, 2018). Vascular endothelial growth factor (VEGF) is a potent angiogenic factor that induces rapid phosphorylation of occludin and ZO-1, disrupting the TJs organization and promoting endothelial permeability (Antonetti et al., 1999; Wang et al., 2001; Storkebaum and Carmeliet, 2004; Murakami et al., 2009). Moreover, pro-inflammatory cytokines, including interferon (IFN)-γ, tumor necrosis factor (TNF)-α and interleukin (IL)-1β, also affect the molecular mechanisms associated with TJs integrity (Capaldo and Nusrat, 2009). Particularly, IFN-γ can directly modify the barrier function of TJs in cultured brain ECs by inducing Rho kinase activity, leading to actin cytoskeletal contractions and junctional disruption (Bonney et al., 2019). In addition, IL-1β reduces the expression of ZO-1 and transendothelial electrical resistance, increasing the paracellular permeability, the expression of adhesion molecules, and leukocyte migration across BBB models *in vitro* (Labus et al., 2014). During neuroinflammation, the release of IL-1β by activated microglia increases the BBB leakage and suppresses the capacity of astrocytes to maintain BBB integrity. Furthermore, IL-1β stimulates astrocytes to produce pro-inflammatory cytokines, promoting BBB breakdown and increased leukocyte migration (Wang et al., 2014). Exposure of brain ECs to pro-inflammatory cytokines also triggers the expression of adhesion molecules, such as P- and E- selectins, VCAM-1 and intercellular adhesion molecule-1 (ICAM-1), which may bind their counter-ligands on activated circulating immune cells, allowing leukocyte extravasation in the inflamed brain (Wong and Dorovini-Zis, 1992; Rahman et al., 2000; Hauptmann et al., 2020). Despite being constitutively expressed on brain ECs, ICAM-1 is further up-regulated in response to pro-inflammatory mediators, and its engagement activates signal transduction pathways leading to increased BBB permeability (Adamson et al., 2002). BBB integrity can also be affected by oxidative stress, particularly by the imbalance between the generation of reactive oxygen species (ROS) and their elimination by scavenger systems. High levels of ROS trigger intracellular pathways leading to TJ rearrangement and matrix metalloproteinase (MMP) activation, which directly compromise BBB integrity. *In vitro* studies demonstrated that H_2_O_2_ induces cytoskeleton dysfunction and alters the localization of occludin and ZO-1, increasing paracellular permeability of brain endothelial cells (Lee et al., 2004; Anasooya Shaji et al., 2019). ROS also enhances the release of MMPs by brain endothelial cells, acting directly on TJ proteins and degrading the extracellular matrix (Aexander et al., 2002; Harada et al., 2012). Large amounts of ROS can be released by parenchymal-resident cells, such as activated microglia, leading to a loss of BBB integrity. Particularly, the microglial NADPH oxidase system is the main producer of superoxide anion (O_2_^–^), which damages ECs and increases BBB permeability (Kahles et al., 2007; Rojo et al., 2014). The principal source of ROS from CNS infiltrating peripheral immune system cells is represented by activated neutrophils, which are professional phagocytes with a strong NADPH oxidase machinery and may also contribute to BBB damage. Neutrophil adhesion to inflamed cerebral vasculature engages integrins leading to ROS production and release of both granule enzymes and complex web-like structures of decondensed DNA fibers and antimicrobial molecules, called neutrophil extracellular traps (NET) (Figure 2; Pietronigro et al., 2017). Thus, migrating neutrophils across the CNS barriers release inflammatory and cytotoxic factors promoting endothelial injury and vascular permeability.

## Neutrophils-blood-brain barrier interplay

Leukocytes receive a variety of signals upon interaction with inflamed endothelial cells, facilitating a cascade of adhesive events, including tethering and rolling, integrin activation, arrest (or firm adhesion), luminal crawling and diapedesis (also called transmigration) (Ley et al., 2007; Vestweber, 2015). Tethering and rolling of circulating leukocytes in inflamed CNS venules are mainly mediated by endothelial P- and E-selectin and their counter-ligands P-selectin glycoprotein ligand-1 (PSGL-1), TIM (T cell immunoglobulin and mucin domain)-1 glycoprotein and CD44 (Siegelman et al., 2000; Piccio et al., 2002, 2005; Battistini et al., 2003; Fabene et al., 2008; Rossi et al., 2011; Angiari and Constantin, 2014; Angiari et al., 2014; Pietronigro et al., 2016; Zenaro et al., 2017; Figure 1). Leukocyte arrest in CNS venules is mediated by the VLA (very late antigen)-4 integrin binding to VCAM-1 and LFA (lymphocyte functional antigen)-1 and Mac-1 integrins binding to ICAM-1 and ICAM-2 (Figure 1; Engelhardt and Ransohoff, 2005; Rossi et al., 2011). Whereas activated T cells mainly rely on VLA-4 (α4β1 integrin) for their intravascular arrest, neutrophils highly depend on β2 integrins (LFA-1 and Mac-1) to arrest and crawl on inflamed brain endothelium (Rossi et al., 2011; Gorina et al., 2014; Zenaro et al., 2015; Pietronigro et al., 2019). However, VLA-4 integrin represents an alternative pathway for neutrophil adhesion, whereas VCAM-1 is a marker of CNS vascular inflammation, suggesting that VLA-4-VCAM-1 interactions may also control neutrophil adhesion during CNS inflammatory diseases (Johnston and Kubes, 1999).

The capacity of neutrophils to increase vascular permeability has been known for four decades, and the interplay between neutrophil adhesion and vascular damage has been studied in several pathological conditions, including brain diseases (Wedmore and Williams, 1981; Wang and Doerschuk, 2002; DiStasi and Ley, 2009; Rossi et al., 2020). In stroke models, neutrophils adhere to cerebral microvessels one hour after stroke induction, suggesting they are early contributors to vascular dysfunction (Hallenbeck et al., 1986; Kataoka et al., 2004; Sienel et al., 2022). Neutrophils can also rapidly adhere in cerebral vessels after status epilepticus induction and contribute to endothelial damage promoting chronic recurrent seizures (Fabene et al., 2008). Interestingly, in both focal ischemia and epilepsy models, it has been suggested that intravascular neutrophil adhesion *per se* without transmigration is sufficient to induce BBB damage and neuronal dysfunction and death, pointing to neutrophil adhesion mechanisms as drug targets for CNS diseases (Fabene et al., 2008; Sienel et al., 2022).

Studies in EAE models also demonstrated the pathogenic role of neutrophils and the contribution of these cells to blood-spinal cord barrier (BSCB) disruption (Wu et al., 2010; Aubé et al., 2014). These data were confirmed by the analysis of postmortem CNS samples of MS patients showing infiltrated neutrophils in areas of BBB or BSCB leakage. However, whereas ICAM-1 and VCAM-1 have been shown to be upregulated on CNS microvascular endothelial cells during EAE, the molecular mechanisms controlling neutrophil adhesive interactions with the BBB or BSCB are still unclear (Steffen et al., 1994). A role for neutrophils has also been shown in AD, and our group has demonstrated that LFA-1 integrin mediates neutrophil interaction with brain ECs expressing ICAM-1 in transgenic mice with AD-like disease (Zenaro et al., 2015). In addition to the adhesion capacity on the vascular wall, neutrophils can also stall in brain capillaries, obstructing blood flow. Indeed, neutrophil depletion induces capillary reperfusion and reduces brain damage in experimental models of stroke and AD (Cruz Hernández et al., 2019; El Amki et al., 2020). Together, these studies suggest a complex detrimental role for neutrophils in CNS inflammatory diseases by increasing vascular permeability following adhesion to the ECs and inducing ischemic phenomena by plugging cerebral capillaries.

During inflammatory responses, neutrophils can release a plethora of factors potentially contributing to tissue injury. Furthermore, it has been known that adhesion on vascular endothelium *via* engagement of β2 integrins leads to neutrophil activation and consequent secretion of inflammation mediators such as ROS and cytotoxic granule enzymes (Figure 2; Richter et al., 1990; Cheung et al., 1993; DiStasi and Ley, 2009). Indeed, in animal models of stroke, NET formation and release of neutrophil elastase (NE) have been reported to induce increased BBB permeability, and the inhibition of these two factors reduces brain injury and promotes recovery (Kang et al., 2020). Neutrophils can also produce MMPs, which have been previously shown to represent a key factor in the induction of vascular damage (Aexander et al., 2002; Kolaczkowska and Kubes, 2013). MMPs were shown to be increased in the CNS during several neuroinflammatory conditions, including MS, stroke, epilepsy, and AD, although their presence was not clearly associated to neutrophil infiltration (Backstrom et al., 1996; Clark et al., 1997; Kieseier et al., 1998; Nygårdas and Hinkkanen, 2002; Solé et al., 2004; Wilczynski et al., 2008; Takács et al., 2010; Quirico-Santos et al., 2013; Py et al., 2014). However, among all MMPs produced by neutrophils, MMP-9 was highly correlated with vascular damage during diseases such as tuberculosis, myocardial infarction and cystic fibrosis (Kurihara et al., 2012; Halade et al., 2013; Garratt et al., 2015; Ong et al., 2017; Gelzo et al., 2022). MMP-9 was also associated with BBB dysfunction, and its expression is involved in the disruption of BBB TJs and degradation of basal lamina (Figure 2; Mun-bryce et al., 1998; Asahi et al., 2001; Moxon-Emre and Schlichter, 2011; Li et al., 2013). Furthermore, MMP-9 expression was clearly correlated with the presence of granulocytes in models of stroke and ischemia-reperfusion injury, in which neutrophils were considered the main source of MMP-9 (Romanic et al., 1998; Gidday et al., 2005; Turner and Sharp, 2016). In addition, by using MMP-9^–/–^ mice, several studies confirmed the critical role of this enzyme in the induction of BBB permeability in stroke models and EAE (Dubois et al., 1999; Asahi et al., 2001; Agrawal et al., 2006; Svedin et al., 2007). Finally, MMP-9-positive neutrophils have been found surrounding brain microvessels with severe type IV collagen degradation and BBB breakdown in patients with stroke, clearly showing a strong association between neutrophils, MMP-9 and BBB damage also during human CNS disease (Rosell et al., 2008). Interestingly, the C-1562T polymorphism in the MMP-9 gene, which is associated with an elevated MMP-9 expression, increases the susceptibility to neuropsychiatric conditions, suggesting that neutrophil MMP-9 may represent a pathogenic factor also in mental disorders (Rybakowski et al., 2009a,b).

Human MMP-9 is covalently linked to lipocalin (LCN2), which increases its stability and confers protection from proteolytic degradation (Tschesche et al., 2001). LCN2 expression is increased during aging and several neuroinflammatory conditions, including AD, MS and stroke suggesting a role for LCN2 in CNS disorders (Anwaar et al., 1998; Marques et al., 2012; Chou et al., 2015; Weng and Chou, 2015; Al Nimer et al., 2016; Dekens et al., 2017). However, whereas *in vitro* data suggested a possible role for LCN2 in preserving BBB integrity, other studies clearly showed that LCN2 expression was found in cerebral endothelial cells and neutrophils in a mouse model of stroke, and its inhibition significantly reduced BBB leakage *in vivo* (Du et al., 2019; Wang et al., 2020). In support of these data, LCN2 was increased in the CNS and CSF in patients with vascular dementia, correlating with reduced expression of TJ proteins and increased BBB permeability, thus suggesting a role for LCN2 in BBB dysfunction (Kim et al., 2017; Llorens et al., 2020).

Myeloperoxidase (MPO) is the most abundant protein stored in neutrophil azurophilic granules. It can be released upon β2 integrin cross-linking in neutrophils and its expression was associated with areas of myeloid cell infiltration in stroke and MS (Figure 2; Matsuo et al., 1994; Walzog et al., 1994; Breckwoldt et al., 2008; Chen et al., 2008; Sajad et al., 2009; Forghani et al., 2012, 2015; Pulli et al., 2015). The link between MPO secretion and endothelial damage has also been reported. Indeed, pharmacological inhibition of MPO attenuates endothelial dysfunction in a mouse model of atherosclerosis (Cheng et al., 2019). Notably, MPO-deficient mice showed decreased leakage in LPS-induced BBB inflammation compared to wild-type littermates (Üllen et al., 2013). These results were supported by *in vitro* data showing that the MPO-H_2_O_2_-Cl_2_ system causes barrier dysfunction in primary brain microvascular endothelial cells by inducing alterations of TJs and AJs (Üllen et al., 2013). Using the MPO inhibitor 4-aminobenzoic acid hydrazide, BBB dysfunction was partially rescued, demonstrating a direct link between BBB increased permeability and MPO release (Üllen et al., 2013). Moreover, in EAE and stroke mouse models, inhibition of MPO with N-acetyl lysyltyrosylcysteine amide prevented BBB breakdown and reduced disease severity, further demonstrating a role for MPO in CNS barrier breakdown (Zhang et al., 2016; Yu et al., 2018). Of note, high expression of MPO was associated with ischemic stroke, and MPO inhibition had beneficial effects, suggesting that MPO may represent a therapeutic target in stroke (Malle et al., 2007; Kim et al., 2016; Kim H. J. et al., 2019; Wang et al., 2022). Interestingly, epidemiological studies associated MPO polymorphisms with an increased risk of neurodegenerative diseases (Reynolds et al., 1999; Crawford et al., 2001; Combarros et al., 2002; Zappia et al., 2004; Pope et al., 2006). Moreover, MPO^+^ cells (probably neutrophils) were increased in the brain in subjects with AD and Parkinson’s disease (PD) (Zenaro et al., 2015; Gellhaar et al., 2017), thus supporting a role for this enzyme in neurodegeneration. Importantly, recent studies by Smyth et al. have shown that MPO staining is mainly associated with infiltrating neutrophils in the AD brain, further providing evidence of a role for neutrophil-derived inflammatory factors in BBB dysfunction (Smyth et al., 2022).

## Choroid plexus: A gateway for neutrophil infiltration

The ChP constitutively expresses adhesion molecules and cytokines supporting continuous immune surveillance of the CNS (Steffen et al., 1996; Meeker et al., 2012; Kunis et al., 2013; Lun et al., 2015; Strominger et al., 2018). Indeed, the stromal compartment of the ChP is populated by several types of cells, including immune cells of peripheral origin (Marques et al., 2012; Schmitt et al., 2012; Szmydynger-Chodobska et al., 2012; Marques and Sousa, 2015). During inflammatory responses, the ChP epithelium and blood vessels further upregulate adhesion receptors and chemokines, facilitating the migration of blood leukocytes into the CSF (Steffen et al., 1994; Vercellino et al., 2008; Marques et al., 2009, 2012; Kunis et al., 2013). In addition to immune cell migration, structural and functional changes at the level of ChP are present during various brain disorders, and BCSFB breakdown may precede clinical symptoms (Schwartz and Baruch, 2014; Alicioglu et al., 2017; Saul et al., 2020; Gião et al., 2022; Mold and Exley, 2022). During AD, the dysfunctional ChP is characterized by alterations of secretory, barrier, transport, and immune functions. Moreover, several imaging studies in AD subjects showed premature ChP aging with increased lipofuscin vacuoles and Biondi bodies compared to age-matched healthy controls (Miklossy et al., 1998; Wen et al., 1999; Serot et al., 2000; Krzyzanowska and Carro, 2012; Tadayon et al., 2020). In addition, the ChP of AD patients have a lower rate of CSF production (Silverberg et al., 2001), show an increase of several inflammatory transcripts and downregulation of claudin-5, claudin-11, and claudin-18, pointing to a role for ChP breakdown in disease development (Bergen et al., 2015; Kant et al., 2018). Expression of TJ proteins in ChP epithelial cells has also been studied in MS. Although the specific functions of individual TJ proteins are unclear, the analysis of post-mortem brain tissues from MS patients showed a selective loss of claudin-3 compared to control tissues. Claudin-3 displays ChP selectivity and is expressed at the apical parts of epithelial cells, acting as a sealant in a similar manner as claudin-5 at the BBB level (Steinemann et al., 2016). Confirming these data, mice lacking claudin-3 showed impaired BCSFB function and a faster onset and increased EAE severity, clearly indicating a role for claudin-3 in ChP breakdown in CNS autoimmune diseases (Kooij et al., 2014).

Neutrophils can migrate into the CSF through a dysfunctional ChP in several CNS inflammatory conditions. Peripheral administration of the TLR2 ligand PAM3CSK4, a prototypic Gram-positive bacterial lipopeptide, induces the migration of neutrophils through the ChP barrier (Mottahedin et al., 2017). Neutrophils can also infiltrate the ChP in EAE mice and represent a source of CSF LCN2 during early disease phases (Figure 1; Marques et al., 2012). Supporting these data, neutrophils migrate into the ChP stroma in MS patients, and this process is probably favored by a high expression of adhesion molecules on ChP blood vessels (Vercellino et al., 2008; Rodríguez-Lorenzo et al., 2020). Similarly, in models of traumatic brain injury (TBI), the choroidal epithelium produces CXCL1 and CXCL2 chemokines, which promote neutrophil migration, supporting the view that ChP represents an entry point for neutrophils invasion of the damaged brain (Figure 1; Szmydynger-Chodobska et al., 2009). Also, neutrophils migrate in the ChP in stroke models, further showing that ChP is a key site for neutrophil infiltration (Otxoa-De-Amezaga et al., 2019). Despite a clear demonstration of neutrophil migration into the ChP during CNS diseases, the molecular mechanisms promoting neutrophil-dependent ChP damage are unclear. However, previous studies have shown a correlation between ChP dysfunction and increased expression of MMP-8 and MMP-9 (Batra et al., 2010). These two enzymes have been shown to mediate neutrophil-dependent damage in other pathological contexts, suggesting this may be the case also during CNS inflammatory conditions. Collectively, these data suggest that ChP is a gateway for neutrophil entry into the brain, and further studies are needed to better understand neutrophil contribution to BCSFB breakdown.

## Neutrophil contribution to meningeal inflammation

The meninges also serve as an important route for immune cell entry into the CNS (Ransohoff et al., 2003; Derk et al., 2021). Indeed, dural venous sinuses were recently shown to be active sites of immune cell trafficking and contain both adaptive and innate immune cells (Mrdjen et al., 2018; Jordão et al., 2019; Van Hove et al., 2019; Rustenhoven et al., 2021). Particularly, recent results have shown that dural sinuses regulate T cell trafficking and contain APCs presenting CSF antigens to patrolling T cells, demonstrating a role for dura in CNS immune surveillance (Rustenhoven et al., 2021). Furthermore, by releasing cytokines, meningeal immune cells regulate several brain functions, including spatial learning (Derecki et al., 2010; Radjavi et al., 2014; Brombacher et al., 2021), short-term memory (Ribeiro et al., 2019), sensory responses (Oetjen et al., 2017) and adult hippocampal neurogenesis (Wolf et al., 2009). Notably, recent studies described meningeal-bone marrow channels directly feeding the meninges with immune cells (Herisson et al., 2018; Brioschi et al., 2021; Cugurra et al., 2021), suggesting that a dysfunctional meningeal immunity may contribute to CNS disorders, such as MS, AD, and PD (Russi and Brown, 2015; Silva and Ferrari, 2019; Zou et al., 2019; Mentis et al., 2021). The meningeal BSCFB is more permissive to the entry of immune cells and large molecules compared to the highly restrictive BBB (Ransohoff et al., 2003). The permeability changes of the arachnoid barrier were less studied in CNS diseases. However, recent studies showed that claudin 11, a TJ protein enriched in the arachnoid barrier, is downregulated in EAE mice (Uchida et al., 2019), indicating a disruption of the arachnoid BCSFB. Increased permeability of the leptomeningeal vessels has also been shown in MRI studies in several brain disorders, including stroke, CNS infections and cerebral amyloid angiopathy (Ineichen et al., 2022). Leptomeninges are now recognized as key players in the development of MS and EAE (Pikor et al., 2015; Russi and Brown, 2015; Rua and McGavern, 2018; Wicken et al., 2018). Indeed, subpial lesions are considered the most common type of lesions in MS patients and autoreactive effector T cells have been shown to infiltrate the leptomeninges via pial microvessels, demonstrating a fundamental immunological role for this vascular district in CNS autoimmunity (Bartholomäus et al., 2009; Schläger et al., 2016; Filippi et al., 2018). Accordingly, reports of gadolinium enhancement in the leptomeningeal compartment have been described in MS, confirming a role for meningeal inflammation in MS (Absinta et al., 2015).

Several studies performed in animal models of AD, MS, TBI, stroke and systemic inflammation have shown increased expression of the adhesion molecules, such as ICAM-1, VCAM-1 and P-selectin, as well as consequent leukocyte adhesion in pial microvasculature (Kerfoot and Kubes, 2002; Piccio et al., 2002; Mel’nikova, 2009; Zhou et al., 2009; Zenaro et al., 2015; Szmydynger-Chodobska et al., 2016; Dusi et al., 2019; Lodygin et al., 2019; Pietronigro et al., 2019). These findings support the idea that meningeal tissue represents a gateway for immune cell access into the CNS and open the possibility of a neutrophil contribution to BCSFB dysfunction (Walker-Caulfield et al., 2015). Indeed, studies performed in patients with stroke and its animal model demonstrated the presence of extravasated neutrophils in the leptomeninges, suggesting a role for neutrophils in meningeal inflammation (Perez-de-Puig et al., 2015; Kim S. W. et al., 2019). Also, in a mouse model of subarachnoid hemorrhage, neutrophils infiltrated the meninges and were associated with neuronal damage (Coulibaly et al., 2021). Notably, mice deficient for MPO lacked neuronal dysfunction suggesting this lysosomal enzyme represents a putative mechanism of neutrophil-dependent damage to the underlying brain tissue in subarachnoid hemorrhage (Coulibaly et al., 2021). Furthermore, studies performed by our group in mouse models of AD showed that neutrophils migrate in pial vessels and brain parenchyma during early disease stages *via* an LFA-1-dependent mechanism promoting cognitive deficit and neuropathological hallmarks of AD (Zenaro et al., 2015). Furthermore, it has been suggested that neutrophil influx in the leptomeninges of EAE mice during pre-clinical stages of the disease promotes early BCSFB breakdown facilitating the access of other immune cells into the CNS (Christy et al., 2013). Overall, although current data provide evidence of neutrophil accumulation in the leptomeninges in models of CNS diseases, how neutrophils promote BCSFB dysfunction and neuronal damage is still largely unknown.

## Conclusion and future directions

The role of neutrophils in the induction of BBB damage is now well documented in animal models of neuroinflammation and patients with CNS diseases. Although less investigated, the contribution of neutrophils to BSCFB breakdown is now emerging from recent studies on stroke, AD and MS. Neutrophil-mediated damage to CNS barriers since early disease stages may pave the way for the recruitment of other leukocyte populations during brain inflammatory diseases, and further studies are needed to clarify this interesting aspect (Christy et al., 2013; Rossi et al., 2020, 2021). However, whereas neutrophil accumulation in the meninges and ChP was clearly demonstrated, the intravascular adhesion mechanisms mediating neutrophil-endothelial interactions and the interplay between neutrophils and other barrier cells, such as ChP epithelial cells, are still unknown. Indeed, *in vivo* tracking of migrating neutrophils in animal models of CNS disorders using advanced microscopy together with non-invasive imaging of radioactive leukocyte tracers may improve our future understanding of neutrophil interaction with CNS borders (Mel’nikova, 2009; Zhou et al., 2009; Szmydynger-Chodobska et al., 2016; Lodygin et al., 2019; Pietronigro et al., 2019). Neutrophils release their arsenal of cytotoxic molecules during inflammatory responses, potentially causing significant collateral tissue damage (Kolaczkowska and Kubes, 2013). However, the molecular mechanisms contributing to neutrophil-dependent inflammation of CNS barriers leading to neuronal and glial cell dysfunction are largely unknown and may represent a new field of investigation. Finally, the elucidation of the molecular pathways underlying neutrophil contribution to CNS diseases may help design new therapies for neurological disorders such as stroke, MS and AD.

## Author contributions

All authors contributed to the literature search and writing the review.
